# Altered White Matter Microstructures in Type 2 Diabetes Mellitus: A Coordinate‐Based Meta‐Analysis of Diffusion Tensor Imaging Studies

**DOI:** 10.3389/fendo.2021.658198

**Published:** 2021-05-03

**Authors:** Cong Zhou, Jie Li, Man Dong, Liangliang Ping, Hao Lin, Yuxin Wang, Shuting Wang, Shuo Gao, Ge Yu, Yuqi Cheng, Xiufeng Xu

**Affiliations:** ^1^ School of Mental Health, Jining Medical University, Jining, China; ^2^ Department of Psychiatry, Jining Psychiatric Hospital, Jining, China; ^3^ Department of Psychiatry, Xiamen Xianyue Hospital, Xiamen, China; ^4^ Department of Psychiatry, The First Affiliated Hospital of Kunming Medical University, Kunming, China

**Keywords:** type 2 diabetes mellitus, diffusion tensor imaging, fractional anisotropy, white matter, meta-analysis

## Abstract

**Objective:**

Type 2 diabetes mellitus (T2DM) is often accompanied by cognitive decline and depressive symptoms. Numerous diffusion tensor imaging (DTI) studies revealed microstructural white matter (WM) abnormalities in T2DM but the findings were inconsistent. The present study aimed to conduct a coordinate‐based meta‐analysis (CBMA) to identify statistical consensus of DTI studies in T2DM.

**Methods:**

We performed a systematic search on relevant studies that reported fractional anisotropy (FA) differences between T2DM patients and healthy controls (HC). The anisotropic effect size seed‐based d mapping (AES-SDM) approach was used to explore WM alterations in T2DM. A meta‐regression was then used to analyze potential influences of sample characteristics on regional FA changes.

**Results:**

A total of eight studies that comprised 245 patients and 200 HC, along with 52 coordinates were extracted. The meta‐analysis identified FA reductions in three clusters including the left inferior network, the corpus callosum (CC), and the left olfactory cortex. Besides, FA in the CC was negatively correlated with body mass index (BMI) in the patients group.

**Conclusions:**

T2DM could lead to subtle WM microstructural alterations, which might be associated with cognitive deficits or emotional distress symptoms. This provides a better understanding of the pathophysiology of neurodegeneration and complications in T2DM.

**Systematic Review Registration:**

Registered at PROSPERO (http://www.crd.york.ac.uk/PROSPERO), registration number: CRD42020218737.

## Introduction

The International Diabetes Federation estimates that 415 million people have diabetes mellitus worldwide, with 90% of these individuals having T2DM (1). Type 2 diabetes mellitus (T2DM) is a chronic metabolic disorder characterized by reduced insulin sensitivity, followed by a compensatory increase in insulin secretion (2). The disease has become a critical health concern worldwide owing to its high prevalence and related disability and mortality (3). T2DM usually leads to various complications in multiple organs, including impairments in the brain (4). People with type 2 diabetes are at an increased risk of cognitive decline and dementia (including Alzheimer’s disease, AD) (5, 6), which is related with worse diabetes management, more frequent occurrence of severe hypoglycemic episodes, and an increased risk of cardiovascular events, and death (7). Earlier meta-analyses showed that the presence of diabetes in older adults was associated with 47% increased risk of all dementia, 39% increased risk of AD, and 138% increased risk of vascular dementia (8, 9). Existing evidence indicated that microstructural brain atrophy contributed to poor cognitive function (10–13). Several neuroimaging studies with different modalities have demonstrated that T2DM is accompanied with structural and functional abnormalities in various regions of the brain (2, 14, 15). Moreover, T2DM and mood disorders share pathophysiological commonalities in the central nervous system (16, 17). The prevalence of depression among T2DM is quite high (18–21), which is considered to be related with cerebral microvascular dysfunction (22). However, the specific neurobiological mechanisms underlying the cognitive impairment and emotional distress of T2DM patients remain unclear for now.

Advances in MRI techniques make it possible to investigate subtle structural alterations of the brain. Among them, diffusion tensor imaging (DTI) is able to detect white matter (WM) microstructure characteristics by estimating random movement of water molecules in the brain (23). The most widely used parameter to study DTI is fractional anisotropy (FA), which reflects diffusion direction and is related to fiber orientation. Any reduction in white matter anisotropy indicates an alteration in the degree of tissue order or integrity (24). DTI approach is widely applied in the evaluation of WM microstructure in various central nervous system disorders. Specially, DTI metrics appears to be a more sensitive marker of cognitive decline due to aging and AD, even when there is no sign of microstructural gray matter (GM) volume alterations and atrophy of brain structures (25, 26). The two most widely used methods of DTI to achieve whole-brain analysis were voxel-based analysis (VBA) and tract-based spatial statistics (TBSS) (27). The former involves analyzing all white matter voxels and correcting for multiple comparisons and noise by reporting only contiguous clusters of significant voxels, while the latter isolates the central core of white matter tracts with the highest FA and reports significant clusters within that white matter skeleton (28, 29). Findings from numerous studies have suggested widespread white matter abnormalities in T2DM patients. However, the results are inconsistent and controversial. According to previous studies, significantly decreased in FA has been observed in patients with T2DM in widespread WM regions such as the frontal lobe (15, 30, 31), temporal region (15, 30–33), corpus callosum (CC) (34–36), cingulum bundle (15, 35, 37), uncinate fasciculus (UF) (35, 36, 38), and corticospinal tract (CST) (35, 36). The inconsistencies of different studies were probably owing to small sample size, heterogeneous demographic characteristics of the patients, and the diversity of methodological techniques.

The coordinate‐based meta‐analysis (CBMA) is a widely used method to solve the discrepancies of regional alterations among various neuroimaging studies (39). The anisotropic effect size seed‐based d mapping (AES-SDM) is an advanced statistical technique for CBMA on different neuroimaging techniques such as structural MRI, functional MRI, DTI, or PET (40). Compared with earlier methods such as activation likelihood estimation and multilevel kernel density analysis (41, 42), the AES‐SDM has strengths as below: (a) In the AES-SDM, both positive and negative differences in the same map are combined to avoid a particular voxel from appearing to be significant in opposite directions (43); (b) The AES-SDM approach allows reported peak coordinates to be combined with statistical parametric maps, thus ensuring more exhaustive and accurate meta‐analyses (44); (c) SDM enables several complementary analyses, such as jack-knife, subgroup, and meta-regression analyses, which can be used to evaluate the robustness and heterogeneity of the results (40). The AES-SDM method has been fully validated in several neuropsychiatric disorders including Parkinson’s disease (45, 46), major depressive disorder (MDD) (29), bipolar disorder (47), obsessive‐compulsive disorder (43, 48, 49), autism spectrum disorder (50), type 1 diabetes mellitus (T1DM) (51), and also in voxel-based morphometry (VBM) studies in T2DM patients (52, 53).

A recently published systematic review of DTI studies (54) comprehensively and systematically summarized previous DTI findings of brain microstructural abnormalities in T2DM. However, this review study is not able to detect the discrepancies of regional alterations with reported coordinates and anisotropic effect size. Thus, a CBMA using AES‐SDM is required to identify consistent results from DTI studies in patients with T2DM. The first objective of this present research was to investigate the most robust FA alterations in T2DM compared with healthy controls (HC). Secondly, we intended to explore the potential effects of demographics and clinical characteristics including mean age, duration of disease, body mass index (BMI), and HbAlc% on WM changes by using meta-regression approach. We hypothesized that patients with T2DM would exhibit microarchitecture alterations in core WM tracts such as the CC, as well as regions related with cognitive functions and emotional regulations.

## Materials and Methods

### Literature Search Strategy

This meta-analysis was conducted according to the Preferred Reporting Items for Systematic Reviews and Meta-Analyses (PRISMA) guidelines (55–57). The protocol of this CBMA was registered at PROSPERO (http://www.crd.york.ac.uk/PROSPERO) (registration number: CRD42020218737). Systematic and comprehensive searches were used to acquire relevant literatures from the PubMed and Web of Science databases published (or “in press”) up to October 31, 2020. The search keywords were (“type 2 diabetes mellitus” or “T2DM” or “type 2 diabetes”) and (“diffusion tensor” or “DTI” or “diffusion magnetic resonance imaging”). Additionally, the reference lists of identified studies and relevant reviews were manually checked to avoid omitting.

### Study Selection

Studies which met the following criteria were included (1): studies compared FA value differences between T2DM and HC in whole-brain analyses (2); reported results in Talairach or Montreal Neurological Institute (MNI) coordinates; (3) used a threshold for significance; (4) articles written in the English language and published in peer-reviewed journals. Exclusion criteria were: (1) meta-analysis, reviews, case reports, or tractography-based only study; (2) studies with no direct between-group comparison; (3) studies from which peak coordinates or parametric maps were unavailable.

### Quality Assessment and Data Extraction

Two authors (ZC and LJ) independently searched the literatures, assessed the quality of the retrieved articles, extracted and cross-checked the data from eligible articles. The quality of the final studies was also independently checked by both authors following guidelines for neuroimaging meta-analyses promoted by Müller and colleagues (58). For each study the following data were recorded: first author, cohort size, demographics (age and gender), illness duration, BMI, HbAlc%, imaging parameters, data processing method and statistical threshold, as well as the three-dimensional peak coordinates of case-control differences in each study.

### AES-SDM Meta-Analysis

Regional FA differences between T2DM patients and HC were performed using the SDM software v5.15 (http://www.sdmproject.com) (43, 59) in a voxel-based meta-analysis approach. We conducted the analysis according to the SDM tutorial and previous meta-analytic studies. The AES-SDM technique uses effect sizes combining with reported peak coordinates which are extracted from databases with statistical parametric maps, and recreates maps of the original maps of the effect size of FA between patients and controls, rather than just assessing the probability or likelihood of a peak (40).

The AES-SDM procedures have been described in detail elsewhere (29, 46, 60),and were briefly summarized as follows: (1) The peak coordinates of all white-matter from each data set were extracted at the level of *t*-statistics (*Z*- or *P*- values for significant clusters which were then converted to *t*-statistics using the SDM online converter); (2) The peak coordinates for each study were recreated using a standard MNI map of the effect size of the group differences in FA by means of an anisotropic Gaussian kernel (44). A relatively wide full width at half maximum (20 mm) and DTI templates were used to control false-positive results; (3) The standard meta-analysis was conducted to create a mean map *via* voxel-wise calculation of the random-effects mean of the study maps. According to Radua et al. (40), an uncorrected *P* = 0.005 using the AES-SDM software is approximately equivalent to a corrected *P* = 0.025. Here, we used more stringent thresholds as follows: uncorrected *P* value < 0.001, peak height threshold *Z* = 1.00, and cluster size threshold = 10 voxels.

### Sensitivity Analyses

To assess the replicability of the results, we performed a systematic whole-brain voxel-based jackknife sensitivity analysis. This procedure involved repeating the main statistical analysis for each result eight times, discarding a different study each time. If a brain region remains significant after running jackknife sensitivity in all or most of the combinations of studies, the finding is considered highly replicable (43).

### Meta-Regression Analysis

Considering the potential influences of mean age, duration of disease, BMI, and HbAlc% on WM abnormalities, a more conservative threshold (*P* < 0.0005) was adopted in consistent with previous meta-analyses and the recommendations of the AES-SDM authors (43), and only brain regions identified in the main effect were considered.

## Results

### Included Studies and Sample Characteristics

**Figure 1:**
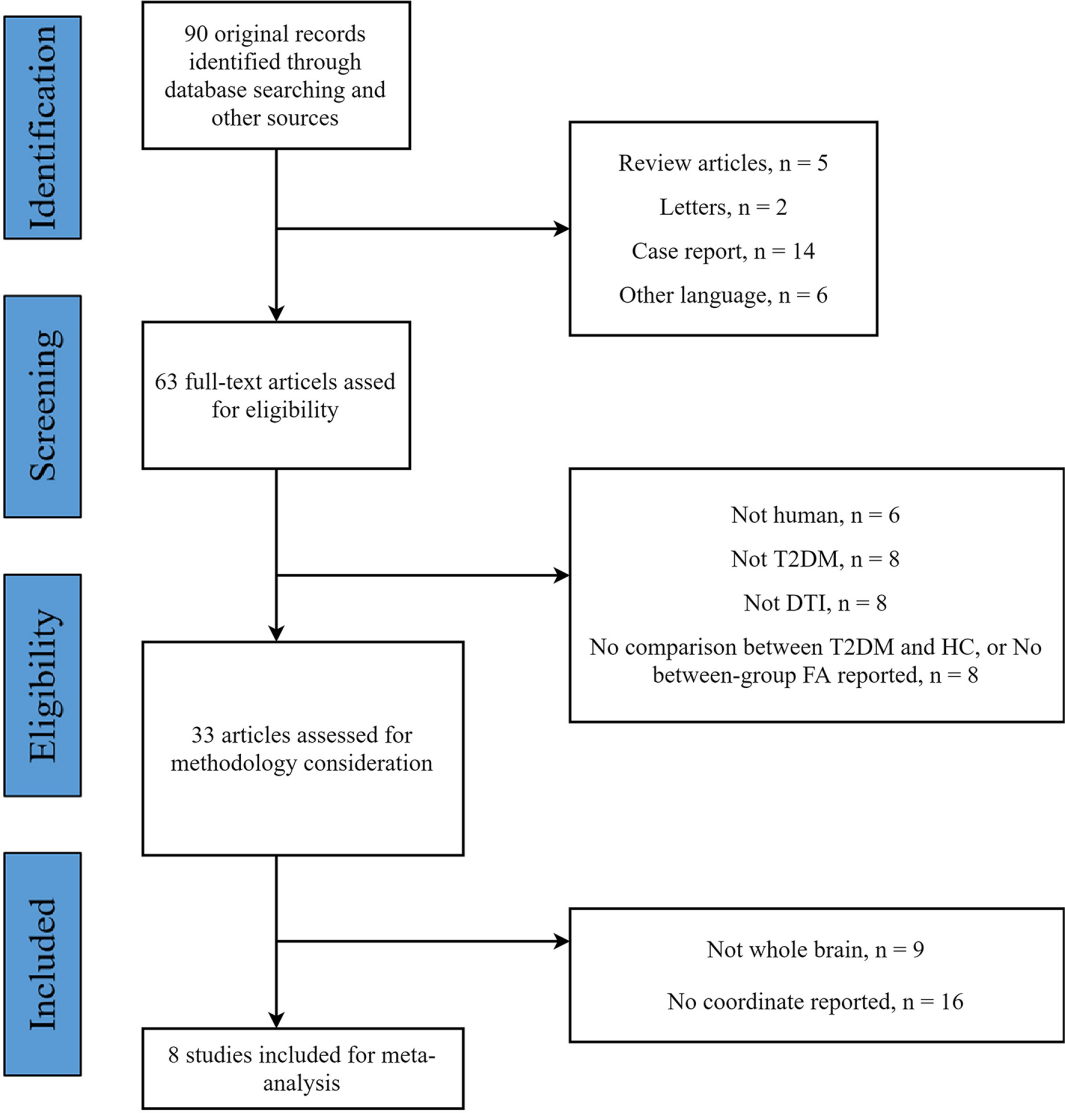
Flow diagram for the identification and exclusion of studies.

The flow diagram of the identification and the attributes of the studies is presented in **Figure 1**. The demographics of the samples are summarized in **Table 1**. The search strategy identified 90 studies, eight of which met the inclusion criteria (15, 30, 32, 34–36, 61, 62). One study contained two different subgroups of T2DM patients (T2DM patients with mild cognitive impairment and T2DM patients with normal cognition), but only the coordinates of significantly different clusters in T2DM patients with mild cognitive impairment were reported. We treated this study as one single dataset. Thus, our final sample comprised 245 T2DM patients and 200 HC, along with 52 coordinates extracted from eight datasets. The scanning methods and FA alterations of the eight datasets are shown in **Table 2**.

**Table 1:** Demographic and clinical characteristics of the participants in eight studies included in the meta-analysis.

Study	Subjects, n (female, *n*)		Age, years		Diabetes duration, years	HbAlc%	BMIkg/m^2^	Comorbidity (number of patients)
	T2DM	HC	T2DM	HC				
Yau et al. (30)	24 (11)	17 (9)	57.2	56.4	7.9	7.8	32.1	Hypertension (16)
Yau et al. (15)	18 (N/A)	18 (N/A)	16.5	17.2	2.6	8.3	37.7	Obesity (18) Hypertension (5)
Kim et al. (34)	20 (11)	20 (11)	54.6	54.3	12.1	10.7	24.7	Diabetic retinopathy (9) Diabetic nephropathy (4) Diabetic peripheral nephropathy (7)
van Bloemendaal et al. (61)	16 (8)	15 (7)	61.4	57.3	7.0	6.9	34.0	Obesity (16)
Nouwen et al. (35)	13 (13)	20 (14)	16.0	16.1	2.6	7.8	N/A	N/A
Yoon et al. (36)	100 (50)	50 (25)	49.2	49.0	1.8	7.1	25.5	Overweight/obesity (50)
Liang et al. (62)	34 (24)	32 (14)	58.3	56.3	6.9	7.9	24.4	Overweight (20) Obesity (1) Hypertension (9)
Xiong et al. (32)	20 (12)	28 (18)	63.6	59.7	9.1	8.2	24.4	Mild cognitive impairment (20)

T2DM, type 2 diabetes mellitus; HC, healthy controls; N/A, not available; BMI, body mass index.

**Table 2:** Scanning methods and FA alterations of the eight studies included in this meta-analysis.

Study	Scanner	Diffusion encoding directions	Type of analysis	Statistical threshold	Number of coordinates	FA alterations
Yau et al. (30)	1.5 T	6	VBA	*P* < 0.005, uncorrected	6	Decrease observed in L temporal stem, R prefrontal region, L frontal temporal region, R external capsule, L parietal region, and L middle temporal region
Yau et al. (15)	1.5 T	6	VBA	*P* < 0.005, uncorrected	3	Decrease observed in R cingulate WM, L cerebral peduncle, and L temporal stem
Kim et al. (34)	3.0 T	30	TBSS	*P <* 0.05, FWE corrected	10	Decrease observed in bilateral posterior thalamic radiation, R retrolenticular part of internal capsule, R splenium of CC, R fornix (cres)/stria terminalis, R sagittal stratum, R external capsule
van Bloemendaal et al. (61)	3.0 T	30	TBSS	*P <* 0.05, FWE corrected	0	–
Nouwen et al. (35)	3.0 T	61	TBSS	*P <* 0.05, TFCE corrected	9	Decrease observed in L CST, medial corpus callosum, L fornix, L thalamic radiation, L retrolenticular internal capsule, L IFOF, R anterior corona radiata, the genu of CC, L uncinate, L callosal body and cingulum, L anterior external capsule, and uncinate fasciculus
Yoon et al. (36)	1.5 T	N/A	VBA	*P* < 0.05, corrected	22	Decrease observed in L fornix sagittal stratum, L IFOF, L uncinate fasciculus, bilateral CST, CC, bilateral anterior thalamic radiation fornix, R superior corona radiata, bilateral cerebellar WM, bilateral forceps minor, bilateral optic radiation, bilateral anterior corona radiata, L external capsule, R parietal WM, and R temporal WM
Liang et al. (62)	3.0 T	25	VBA	*P* < 0.05, AlphaSim corrected	1	L corona
Xiong et al. (32)	3.0 T	25	TBSS	*P <* 0.05, FWE corrected	1	R temporal lobe

CC, corpus callosum; CST, corticospinal tract; FA, fractional anisotropy; FWE, family-wise error; IFOF, inferior fronto-occipital fasciculus; N/A, not available; L, left; R, right; T, Tesla; TBSS, tract-based spatial statistics; TFCE, threshold-free cluster enhancement; VBA, voxel-based analysis; WM, white matter.

### Regional Differences in FA

**Figure 2:**
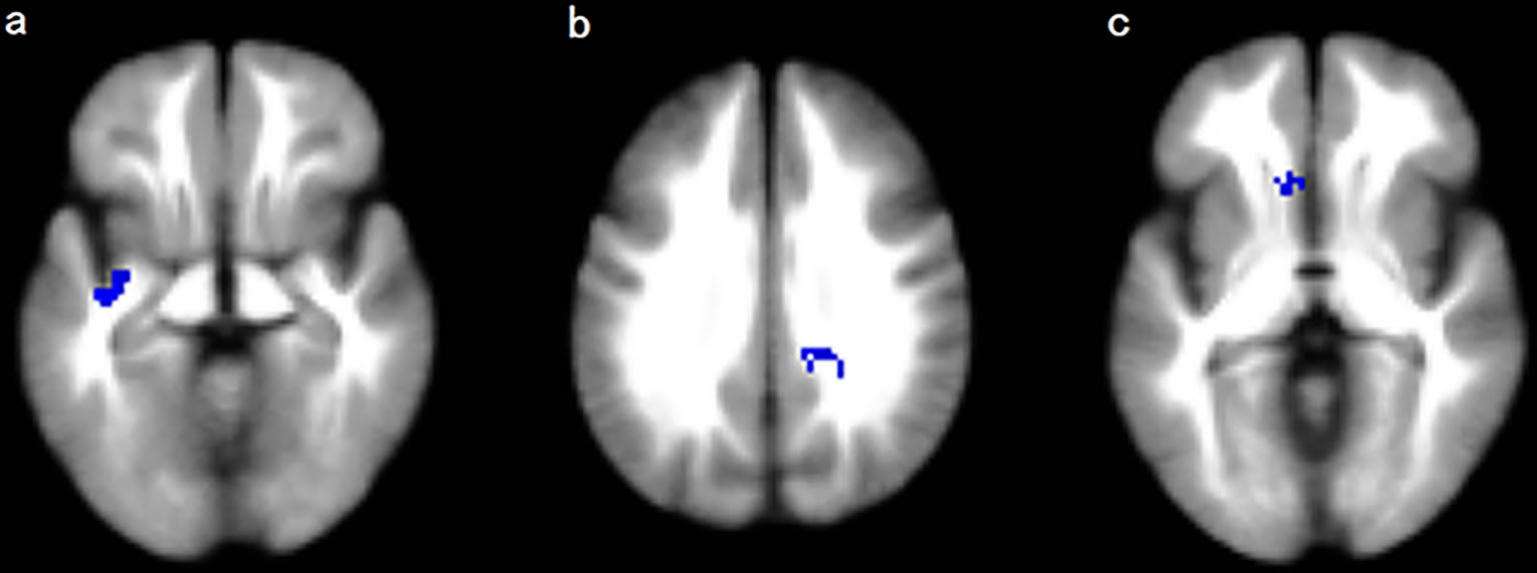
Regions showing FA reductions in **(A)** the left inferior network; **(B)** the corpus callosum; and **(C)** the left olfactory cortex. Significant clusters are overlaid on MRIcron template for Windows for display purposes only.

The meta-analysis revealed that patients with T2DM exhibited significant FA reductions in three clusters relative to HC, including the left inferior network, the CC and left olfactory cortex (BA 25), as illustrated in **Figure 2** and **Table 3**. No region with higher FA was identified in the current meta‐analysis.

**Table 3:** White Matter Regions of FA reductions in T2DM Patients compared to healthy controls in the coordinate-based meta-analysis.

Regions	Maximum					Cluster		Jackknife sensitivity analysis
	MNI coordinates			SDM Value	*P*	Number of voxels^*^	Breakdown (number of voxels)	
	X	Y	Z					
Left inferior network, inferior fronto-occipital fasciculus	−38	−16	−10	−2.279	0.000032604	97	Left inferior network, inferior fronto-occipital fasciculus (28) Left inferior network, inferior longitudinal fasciculus (20) Anterior commissure (8) Left inferior network, uncinate fasciculus (7) Left insula, BA 48 (2) Left amygdala, BA 34 (1) Left superior temporal gyrus, BA 48 (1) BA 48 (20) BA 34 (3) BA 36 (3) BA 20 (3) BA 21 (1)	7/8
Corpus callosum	14	−34	32	−2.107	0.000091314	55	Corpus callosum (46) Right median network, cingulum (9)	7/8
Left olfactory cortex, BA 25	-4	20	4	−1.999	0.000228226	28	Corpus callosum (12) Left striatum (9) Left caudate nucleus, BA 25 (3) Left olfactory cortex, BA 25 (3) BA 25 (1)	6/8

^*^All voxels with P < 0.001 uncorrected.

BA, Brodmann area; FA, fractional anisotropy; MNI, Montreal Neurological Institute; SDM, seed‐based d mapping; T2DM, type 2 diabetes.

### Jackknife Sensitivity Analysis

The whole-brain jackknife sensitivity analysis revealed that decreased FA in T2DM patients in the left inferior network and the CC was highly replicable, as these findings were preserved throughout all but one combination of the datasets. FA reduction in the left olfactory cortex remained significant in all but two combinations (**Table 3**).

### Meta-Regression Analysis

At a stringent threshold of *P* < 0.0005, meta-regression analysis found a negative correlation between FA in the CC and BMI in the patients group (**Table 4**). The mean age of patients, illness duration, and HbAlc% were not linearly associated with FA changes.

**Table 4:** Correlation between FA alterations and BMI in T2DM revealed by Meta‐regression analyses.

Factor	Anatomic label	MNI coordinates			SDM Value	*P*	Number of voxels
		X	Y	Z			
BMI	Corpus callosum	14	−32	30	−2.390	0.000045657	58

BMI, body mass index; FA, fractional anisotropy; MNI, Montreal Neurological Institute; SDM, seed‐based d mapping; T2DM, type 2 diabetes.

## Discussion

To our knowledge, this study is the first coordinate‐based meta-analysis (CBMA) of DTI studies in T2DM patients investigating microstructural WM abnormalities and examining how clinical features affect WM morphometry. Using the AES-SDM meta-analytical approach, this study identified decreased FA in three clusters, and these three regional differences remained replicable in the Jackknife sensitivity analyses. The largest cluster exhibited a peak coordinate in the left inferior network mainly consisted of left inferior fronto-occipital fasciculus (IFOF), left inferior longitudinal fasciculus (ILF), left uncinate fasciculus (UF), and anterior commissure. Other clusters exhibited FA reductions in the CC and the left olfactory cortex (BA25). Besides, according to the meta-regression, FA in the CC was negatively correlated with BMI in the patients group. These findings enhanced our understanding of the underlying neurodegeneration in T2DM.

Our meta‐analysis only identified lower FA rather than higher FA in T2DM patients. This is in accordance with most published DTI studies of T2DM (54). As FA presents the anisotropic diffusion of water molecules and can reflect the underlying characteristics of microstructure, such as fiber density, axonal diameter, thickness of the myelin sheaths, and directionality of the fibers (27, 63), decreased FA in our findings represented disrupted WM microarchitecture in the brain. One of the core characteristics of T2DM is insulin resistance, which interferes with glucose metabolism and even can lead to increased plasma glucose in regional brain areas in T2DM patients (52). From the microscopic point of view, hyperglycemia is considered to be related with various metabolic and molecular alterations and could result in brain cell dysfunction, degeneration, or death ultimately (52, 64). And from the macroscopic perspective, brain atrophy might be the neurobiological basis of cognitive decline (5, 6, 11). This was also in agreement with previous VBM meta‐analyses of T2DM (52, 53).

The left inferior network mainly comprised the left IFOF, left ILF, left UF, and anterior commissure. Several studies support the extension of WM impairments in T2DM to other association fibers, which pass through the temporal lobe, such as IFOF and ILF (33, 36, 54). Besides, some fibers of IFOF and UF are located in the external capsule, which associates the hippocampus and amygdala with prefrontal and orbitofrontal cortices (54, 65). Previous studies already indicated that atrophy in temporal lobe, hippocampus, and orbitofrontal regions occurred in T2DM (2, 13, 54), and also evidence has shown that atrophy in these areas is one of the earliest neuroanatomical changes in Alzheimer’s dementia (2, 36, 53). Among the eight studies included in our meta-analysis, four of them reported microstructural abnormalities in temporal regions. Given that the vital role that the temporal lobe, the hippocampus, and the orbitofrontal cortex play in cognitive processes such as learning, memory, and decision making (66, 67), we conjectured that disruptions of WM in IFOF, ILF, and UF might be related with cognitive function deficits in T2DM patients. Besides, the comorbidity of depression and T2DM is quite common (18–21), and disrupted WM connectivity in inferior network has also been constantly found in MDD patients (29, 68, 69). Thus, microarchitecture alterations in the inferior network might also underlie potential affective changes in T2DM.

The CC is the largest interhemispheric WM commissure connecting the cerebral hemispheres, and plays crucial role in interhemispheric communication and cognitive processes (70). Microstructural changes in this core WM tract were found not only in T2DM patients in numerous research (34–36, 38, 54), but also in patients with cognitive impairment (38, 71, 72) and patients with MDD (29, 68, 73, 74). Therefore, decreased FA in the CC observed in our meta-analysis may underlie the deficits in cognitive processing and emotional modulation in patients with T2DM. Besides, there was a negative correlation between FA in the CC and BMI in T2DM patients revealed by meta-regression analysis. This was consistent with previous findings that higher BMI was associated with FA reductions in the CC in healthy cohorts (75, 76). There were DTI studies on BMI-related WM abnormalities suggesting a primordial effect of BMI on brain circuits involved in reward processing and emotion regulation (77), or even on the entire brain (75). Furthermore, there was evidence that alterations in white matter were associated with several obesity-related conditions such as cardiovascular risk factors including metabolic syndrome (78). Therefore, our finding might suggest disrupted CC microstructures as an BMI-related neurobiological marker of T2DM. The other WM tracts showed non-significant regression results, probably due to a relatively strict *P*-value in the process of statistics. Neurologic changes in the left inferior network and the left olfactory cortex might also be associated with metabolic syndrome related symptoms and these regions should receive full considerations.

It is particularly noteworthy that the left olfactory cortex exhibited decreased FA in T2DM patients. Current evidence implied that olfactory function is associated with the emergence of prodromal AD (79, 80). Scholars assumed that olfactory impairments might reflect the onset of AD, amnestic mild cognitive impairment (MCI), and the presence of amyloid-β (Aβ) and tau pathology (79, 81–86). Thus, FA reductions in the left olfactory cortex might be served as an early prediction of cognitive impairment in T2DM patients. This was of great significance for early detection of potential cognitive decline and dementia in T2DM patients. Moreover, olfactory function was also found to be related to the pathogenesis of MDD (87). Olfactory sulcus structural abnormality might be a trait-related marker of vulnerability to MDD (88). In consideration of the high prevalence of comorbidity of depression and T2DM, olfactory cortex alterations might be involved in the pathophysiology of the co-morbidity.

Several limitations of this study should be noted. Firstly, as the number of studies included in our meta-analysis was small, we were not able to perform separate subgroup meta-analyses for clinical variables such as cognition status, depression severity, and BMI, or methodological differences such as VBA and TBSS, which would likely diversify the results. Secondly, the data acquisition parameters, participants characteristics and clinical variables in the included studies were heterogeneous. It is not possible to eliminate these differences by statistical means. Thirdly, our analysis was limited to WM diffusion changes thereby not including the large amount of research on GM volume or WM volume. Future meta-analysis could include VBM studies for a more comprehensive perspective of the brain microarchitecture. Last but not least, it is meaningful to work on the reversibility of nerve damage, but the present meta-analysis and the literatures included in our research are all cross-sectional design. Longitudinal studies with respect to reversibility of the neurodegeneration of T2DM is of great importance and should be addressed in the future.

## Conclusion

The present meta-analysis indicated that T2DM patients demonstrated significant FA reductions in the left inferior network, the CC and the left olfactory cortex. Among them, FA of the CC had a negative correlation with BMI in the patients group. These findings supported the opinion that T2DM could lead to subtle WM structural alterations, which might be associated with cognitive deficits or emotional distress in T2DM patients. This helps us better understand the neural mechanism underlying neurodegeneration in T2DM.

## DATA AVAILABILITY STATEMENT

The original contributions presented in the study are included in the article/supplementary material. Further inquiries can be directed to the corresponding author.

## Author contributions

CZ designed the study and revised the manuscript. CZ wrote the initial manuscript. CZ and JL collected the data and undertook the statistical analysis. MD and LP assisted with data collection and statistical analysis and modified the paper. HL, YW, SW, SG, and GY assisted with data collection and data analysis. YC and XX critically reviewed and modified the paper. All authors contributed to the article and approved the submitted version.

## FUNDING

This study was supported by the Medical and Health Science and Technology Development Plan of Shandong Province (202003061210), and the Supporting Fund for Teachers’ Research of Jining Medical University. The Supporting Fund for Teachers’ Research of Jining Medical University (600903001).

## Conflict of Interest

The authors declare that the research was conducted in the absence of any commercial or financial relationships that could be construed as a potential conflict of interest.
